# Determining the communicable period of SARS-CoV-2: A rapid review of the literature, March to September 2020

**DOI:** 10.2807/1560-7917.ES.2021.26.14.2001506

**Published:** 2021-04-08

**Authors:** Mina Park, Colleen Pawliuk, Tribesty Nguyen, Amanda Griffitt, Linda Dix-Cooper, Nadia Fourik, Martin Dawes

**Affiliations:** 1 Vancouver Coastal Health, Vancouver, British Columbia, Canada; 2 School of Population and Public Health, University of British Columbia, Vancouver, British Columbia, Canada; 3 School of Information, University of British Columbia, Vancouver, British Columbia, Canada; 4 Faculty of Medicine, University of British Columbia, Vancouver, British Columbia, Canada; 5 Department of Family Practice, University of British Columbia, Vancouver, British Columbia, Canada

**Keywords:** COVID-19, SARS-CoV-2, transmission, communicable, duration, rapid review

## Abstract

**Introduction:**

Standard testing for infection with severe acute respiratory syndrome coronavirus 2 (SARS-CoV-2) is based on RT-PCR tests, but detection of viral genetic material alone does not indicate ongoing infectious potential. The ability to isolate whole virus represents a better proxy for infectivity.

**Aim:**

The objective of this study was to gain an understanding of the current literature and compare the reported periods of positive SARS-CoV-2 detection from studies that conducted RT-PCR testing in addition to experiments isolating whole virus.

**Methods:**

Using a rapid review approach, studies reporting empirical data on the duration of positive RT-PCR results and/or successful viral isolation following SARS-CoV-2 infection in humans were identified through searches of peer-reviewed and pre-print health sciences literature. Articles were screened for relevance, then data were extracted, analysed, and synthesised.

**Results:**

Of the 160 studies included for qualitative analysis, 84% (n = 135) investigated duration of positive RT-PCR tests only, 5% (n = 8) investigated duration of successful viral isolations, while 11% (n = 17) included measurements on both. There was significant heterogeneity in reported data. There was a prolonged time to viral clearance when deduced from RT-PCR tests compared with viral isolations (median: 26 vs 9 days).

**Discussion:**

Findings from this review support a minimum 10-day period of isolation but certain cases where virus was isolated after 10 days were identified. Given the extended time to viral clearance from RT-PCR tests, future research should ensure standard reporting of RT-PCR protocols and results to help inform testing policies aimed at clearance from isolation.

## Introduction

Understanding how long individuals may continue to transmit virus after infection with severe acute respiratory syndrome coronavirus 2 (SARS-CoV-2) is important to inform testing policies and isolation procedures required to prevent nosocomial and community spread. Numerous studies have been conducted to date to address this question; however, the literature has been predominantly represented by studies using methods based on reverse transcription-PCR (RT-PCR) tests. As RT-PCR tests detect the presence of viral genetic material and thus do not differentiate between live (or viable) and non-infective virus [1], inferences from these results as to potential infectious periods are limited.

A more accurate proxy of infectious potential is based on the ability of whole virus to be successfully isolated and cultured in laboratory settings, with clinical confirmation of transmission where possible. While fewer studies have been conducted that have successfully isolated and cultured live virus, results from this literature have contributed to a number of reviews [2-7]. The findings from these reviews have drawn consistent conclusions that align with previously recommended isolation strategies [8]: overall, infectious potential appears to be greatly reduced by day 10 following symptom onset of coronavirus disease (COVID-19) [2,7,9].

However, a growing concern relates to individuals who continue to test positive by RT-PCR over extended periods of time [10-16], including those who re-test positive after an initial negative test result [10]. As RT-PCR tests of respiratory samples have been and will remain the de facto method of confirming initial and ongoing infection with SARS-CoV-2, it is important to be able to interpret positive test results that are obtained throughout the disease course, including during convalescence. Against a backdrop of dramatically rising cases internationally, limited testing resources and public health capacity for ongoing case management, and ongoing restrictions on mobility, there is a need for as much evidence as possible to help understand the likely implication of ongoing positive tests or re-tests after cessation of the recommended period of isolation, on potential disease spread.

The purpose of this review was to conduct a rapid review of existing literature in order to directly compare the duration of potential infectivity of SARS-CoV-2 from studies that obtained measurements of the duration of infectious potential using both RT-PCR and viral isolation methods. An additional, and related, objective was to understand and be able to provide an overview of the literature at the time the review was conducted (until end of September 2020), especially given the evolving nature of the pandemic.

## Methods

We conducted a rapid review of the literature using the methods outlined in the National Collaborating Centre for Methods and Tools Guidebook [17]. We used the Preferred Reporting Items for Systematic Reviews and Meta-Analyses (PRISMA) guidelines to report our rapid review [18]. We did not publish or pre-register a protocol for our review given time pressures for this information. From the available data, duration (maximums, medians or means values) was retrieved or calculated. Ethics approval was not required.

### Search strategy 

Databases of peer-reviewed and pre-print health sciences literature (Ovid MEDLINE, Embase, Google Scholar, medRxiv and arXiv) and the grey literature for reports or guidelines on discontinuation of isolation for SARS-CoV-2 from international and national public health organisations (World Health Organization, European Centre for Disease Prevention and Control, United States Centre for Disease Control websites) were searched using two search strategies: (i) terms for ‘SARS-CoV-2’ and ‘viral clearance/shedding,’ on 23 May 2020 and (ii) terms for ‘SARS-CoV-2’ and ‘viral isolation/culture,’ on 1 July 2020. The latter search was done with specific terms because there was a paucity of studies with data from viral isolation/cultures identified in the previous iteration of the search; moreover, in order to include more recently published studies, we examined other reviews, including a living evidence review, until 29 September 2020 and added relevant references to our results [1,2,5-7,9]. All databases were searched from inception and searches were limited to English. Detailed information on search strategies undertaken in each database can be found in the Supplement (Appendix A). Additional studies were identified by reviewing the references of select high-impact articles, reports from reputable sources, and existing reviews. Any studies identified through other sources or contacts were manually added.

### Inclusion criteria

We included studies presenting primary empirical data on duration of possible infectivity of SARS-CoV-2 in human populations using respiratory samples and reported in English. Articles that did not report data on duration of potential infectiousness in text, figures or tables were excluded. Studies reporting solely on pre-symptomatic or on convalescent period durations, incubation periods, serial intervals or on results based on statistical modelling were excluded. Studies, including reviews, that used data from other investigations were excluded; where relevant studies were identified, their references were reviewed to add relevant primary studies. When duplicate study reports were identified (i.e. a pre-print and a peer-reviewed journal article), the most recent version was included. Inclusion criteria are outlined in more detail in the Supplement (Appendix B).

### Screening process

Titles and abstracts were screened for relevance independently by two reviewers. Full-text review was conducted independently by two reviewers for articles where relevance was not readily determined from title and abstracts, and any conflicts were resolved through consensus by the two reviewers.

### Data extraction process

A draft data extraction form was developed and trialled across multiple reviewers to develop the final version. Extracted data fields included study characteristics (first author, publication status, study type, sample size), study population characteristics (age, hospitalisation, disease severity), method of determining infectious period (viral shedding, viral isolation), type of respiratory specimen(s) collected, reported durations (minimum, mean, median, maximum), whether cases without symptoms (asymptomatic or pre-symptomatic) were reported, whether the study focused solely on duration of communicability during the convalescent phase, how measurement of duration start and end was defined, and study quality. All extracted data were reviewed by a second reviewer.

### Definitions

Sample size was defined as the total number of participants for whom data on communicability period was assessed. Disease severity associated with SARS-CoV-2 infection was classified according to the following definitions: mild referred to study populations reporting no symptoms or non-serious symptoms that did not require healthcare intervention, moderate severity included participants who required acute care and/or intervention, and severe disease referred to cases that required admission to the intensive care unit, critical intervention and/or resulted in death. Studies that included participants with mild, moderate and severe cases were classified as ‘mixed’; otherwise, if they included cases that were mild/moderate or moderate/severe, they were categorised as the higher level of severity. Hospitalisation status was determined as described in the study. Notably, in some jurisdictions, admission to hospital appeared to be part of routine isolation policies and so this description alone was not taken as an indicator of disease severity. Studies with children were those that included participants aged 19 years or younger. Respiratory samples included those taken from the upper (naso/oro-pharyngeal, nasal, throat, or saliva swabs) or lower respiratory tract (from sputum or bronchial lavage specimens). For the purposes of this review, the start of measurement of duration is referred to as ‘symptom onset’ and measurement end as ‘viral clearance’. The end point for measuring duration of viral isolation was the last reported day on which virus could be isolated and cultured (captured under ‘other’).

### Assessment of study quality

An adaptation of the Mixed Methods Appraisal Tool was used to assess study quality [19]. Questions were concerned with the following: (i) the study had clear research questions or objectives, (ii) the collected data allowed the study to address the stated research question, (iii) the research question was aimed at understanding duration of communicability, (iv) there was a complete follow-up period defined to measure duration of communicability and (v) there was clarity about when measurement of communicability period started, about sample types collected and frequency of sample collection, about how long patients were followed (until viral clearance, study end, hospital discharge, death) and about the method of assessment of communicability. Owing to the emerging nature of this topic, we did not exclude studies from our synthesis or analysis based on study quality.

### Analysis

All data processing and analysis was conducted using the statistical programming language R (version 4.0.0) [20]. For studies where more than one value was reported for duration (i.e. when data for multiple sample types were reported or results were presented in a stratified manner), the values corresponding to the higher duration were included for analysis. As analyses were generally aimed at identifying maximum reported durations, these values were pulled from each study and summarised. Raw data are available in Supplementary Table S1.

## Results

### Results of literature search

**Figure 1:**
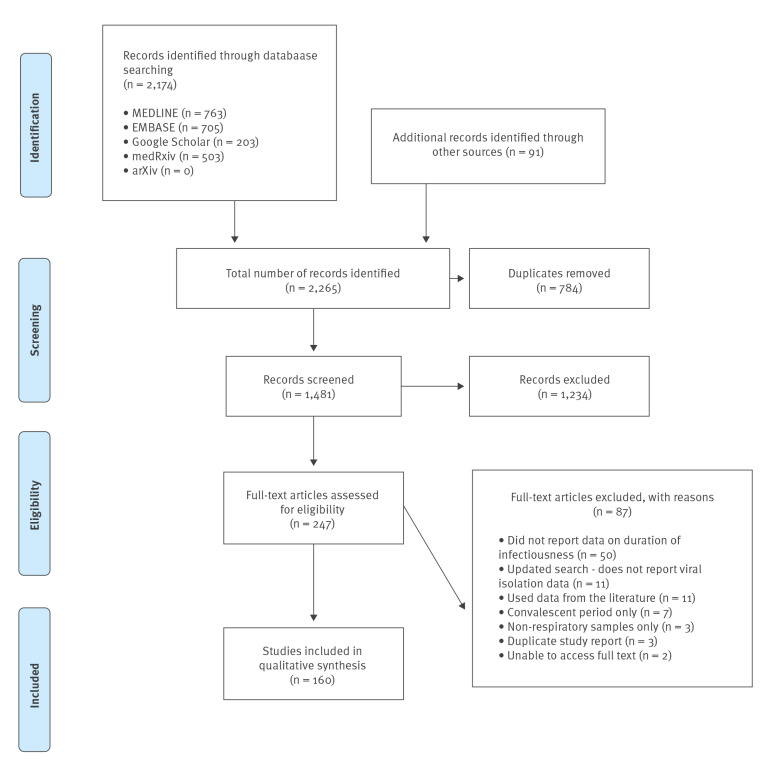
PRISMA flowchart diagram of included studies on the communicable period of SARS-CoV-2, March–September 2020 (n = 160)

We retrieved, 2,174 records from database searches and 91 additional studies from reference chaining and other sources (2,265 total), of which 1,481 remained after removing duplicate records. Of these, 1,234 were excluded in screening and 87 in full-text review as they did not report data on duration of potential infectivity, used secondary data from the literature, reported only on the convalescent period, assessed non-respiratory samples only, the full text was not accessible, or they were duplicate reports of the same study. 160 studies were included in the final synthesis. The PRISMA flowchart in Figure 1 illustrates the study selection process.

SARS-CoV-2: severe acute respiratory syndrome coronavirus 2.

The PRISMA flowchart details the number of studies identified through our search and then included for analysis after screening for eligibility [18].

### Study characteristics

#### Study populations

Information on study characteristics is presented in Table 1, overall and broken down by method of assessment. Of 160 total included studies, 17 studies assessed duration through both viral isolation and RT-PCR, while eight focused on viral isolation only and 135 included only results from RT-PCR. There was a wide range of study sample sizes (between one and 1,061), with a mean of 58.5 (standard deviation (SD): 118.9) and a median of 16.5 (interquartile range (IQR): 68.5). The majority of studies consisted of case series (n = 101; 63%), while 35 (22%) were case reports, cohort (n = 15; 9%), cross-sectional (n = 4; 3%), clinical trial (n = 3; 2%) and case control studies (n = 2; 1%) (Table 1). The results of the study quality assessment are included in Table 1.

**Table 1:** Characteristics of included studies, overall and broken down by method of assessment of duration of communicability of SARS-CoV-2, March–September 2020 (n = 160)

	All studies (n = 160)		Viral isolation only (n = 8) [38-44,46]		RT-PCR only (n = 135) [12-16,48-177]		Viral isolation and RT-PCR (n = 17) [21-37]	
Sample size								
Minimum	1		5		1		1	
Maximum	1,061		518		584		1,061	
Mean (SD)	58.5 (118.9)		142.5 (167.8)		49.3 (83.9)		92 (252.3)	
Median (IQR)	16.5 (68.5)		97 (153.3)		14 (64.5)		12 (75.0)	
Study design	n	%	n	%	N	%	n	%
Case control	2	1.3	0	0.0	2	1.5	0	0.0
Case report	35	21.9	0	0.0	30	22.2	5	29.4
Case series	101	63.1	7	87.5	84	62.2	10	58.8
Clinical trial	3	1.9	0	0.0	3	2.2	0	0.0
Cohort	15	9.4	0	0.0	15	11.1	0	0.0
Cross-sectional	4	2.5	1	12.5	1	0.7	2	11.8
Measurement start	n	%	n	%	n	%	n	%
Hospital admission	36	22.5	0	0.0	33	24.4	3	17.7
Date of first positive test	12	7.5	1	12.5	11	8.1	0	0.0
Symptom onset	91	56.9	7	87.5	72	53.3	12	70.6
Other	21	13.1	0	0.0	19	14.1	2	11.8
Measurement end	n	%	n	%	n	%	n	%
Negative test	114	71.3	3	37.5	105	77.8	7	41.2
Discharge/death	8	5.0	0	0.0	7	5.2	1	5.9
Last positive test	7	4.4	2	25.0	3	2.2	2	11.8
Other	31	19.4	3	37.5	20	14.8	7	41.2
Region	n	%	n	%	n	%	n	%
Asia	127	79.4	1	12.5	119	88.1	7	41.2
Australia	2	1.3	1	12.5	1	0.7	0	0.0
Europe	19	11.9	5	62.5	8	5.9	6	35.3
Middle-East	2	1.3	0	0.0	2	1.5	0	0.0
North America	10	6.3	1	12.5	5	3.7	4	23.5
Age group	n	%	n	%	n	%	n	%
Children	18	11.3	2	25.0	16	11.9	0	0.0
Adults	114	71.3	4	50.0	97	71.1	14	82.4
Mixed	28	17.5	2	25.0	23	17.0	3	17.6
Hospitalisation status	n	%	n	%	n	%	n	%
Hospitalised	136	85.0	3	37.5	119	88.1	14	82.4
Not hospitalised	4	2.5	0	0.0	4	3.0	0	0.0
Mixed	17	10.6	5	62.5	10	7.4	2	11.8
Unclear	3	1.9	0	0.0	2	1.5	1	5.9
Disease severity	n	%	n	%	n	%	n	%
Mild	36	22.5	0	0.0	33	24.4	3	17.7
Moderate	32	20.0	0	0.0	29	21.5	3	17.7
Severe	12	7.5	1	12.5	10	7.4	1	5.9
Mixed	51	31.9	6	75.0	38	28.1	7	41.2
Unclear	29	18.1	1	12.5	25	18.5	3	17.7
Includes asymptomatic or pre-symptomatic cases	n	%	n	%	n	%	n	%
Yes	69	43.1	2	25.0	62	45.9	5	29.4
No	91	56.9	6	75.0	73	54.1	12	70.6
Publication status	n	%	n	%	n	%	n	%
Peer-reviewed journal	134	83.8	5	62.5	112	83.0	17	100.0
Preprint database	26	16.3	3	37.5	23	17.0	0	0.0
Study quality	n	%	n	%	n	%	n	%
0 quality concerns	31	19.4	3	37.5	26	19.3	2	11.8
1 quality concern	43	26.9	1	12.5	36	26.7	6	35.3
2 quality concerns	53	33.1	4	50.0	44	32.6	5	29.4
3 quality concerns	27	16.9	0	0.0	23	17.0	4	23.5
4 quality concerns	4	2.5	0	0.0	4	3.0	0	0.0
5 quality concerns	2	1.3	0	0.0	2	1.5	0	0.0

IQR: interquartile range; SARS-CoV-2: severe acute respiratory syndrome coronavirus 2; SD: standard deviation.

The majority of studies were conducted in Asia (n = 127, 79%), with 19 studies conducted in Europe (12%), 10 in North America (6%), two in the Middle East (1%) and two in Australia (1%) (Table 1). Study populations had mixed disease severity (i.e. included mild, moderate and severe disease) in 51 studies (32%), and 29 studies (18%) did not provide information on disease severity. Thirty-six studies (23%) had participants with only mild symptoms, while 32 studies (20%) studied moderate symptoms and 12 (8%) severe disease. The majority of studies included only hospitalised patients (n = 136; 85%). Most of the studies included only adults (n = 114; 71%), while 18 studies (11%) focused solely on paediatric populations, and 28 studies (18%) had a mixed population of children and adults. Sixty-nine studies (43%) included asymptomatic or pre-symptomatic positive cases (Table 1).

#### Measurement of duration start and end

The starting point for measuring duration was primarily symptom onset (n = 91; 57%), followed by hospital admission (n = 36; 23%), when the patient first tested positive for SARS-CoV-2 (n = 12; 8%) or other (n = 21; 13%). The end point of duration measurement for most studies was the date of a single or consecutive negative RT-PCR test (n = 114; 71%), followed by discharge/death (n = 8; 5%), last positive test (n = 7; 4%) or other (n = 31; 19%). 

#### Study quality

At the time of writing, 26 (16%) of the studies included in this review were pre-prints and thus had not undergone peer-review. Many of the remaining studies published in peer-review journals were letters to the editor or other short communications that do not fully report their methods. There was variation in study quality in both preprints and studies published in peer-reviewed journals; roughly half of the included studies (n = 86; 54%) had two or more study quality concerns (Table 1).

### Overview of studies that measured durations using both RT-PCR tests and viral isolation experiments

There were 17 studies that investigated the duration of time until viral clearance through RT-PCR tests as well as viral isolation [21-37]. Eight other studies investigated viral isolation durations only [36,38-44]. Supplementary Table S2 displays detailed information on each individual study.

Table 2 provides an overview of the 12 studies that included measurements of the maximum time to viral clearance from both RT-PCR and viral isolation experiments. The studies included mixed clinical populations and determined the duration of viral viability either by taking a cross-section of diagnostic samples collected at different times from symptom onset (n = 2; 17%) or by serially collecting samples from the same individuals over time (n = 10; 83%). While not all studies reported the RT-PCR cycle threshold (Ct) values of samples that achieved viral isolation, of those that did, the range of Ct values with a successful isolation was 12.3–37.9.

**Table 2:** Overview of studies that included measurements of duration to SARS-CoV-2 clearance from both RT-PCR and viral isolation experiments, March–September 2020 (n = 12)

Reference	Study population description	Sample size	Sample types taken for isolation and sampling method	Longest time to viral clearance (RT-PCR) (in days)	Longest time to viral clearance (isolation) (in days)	Ct values
Arons et al. [22]	Patients in a skilled nursing facility with mixed disease severity; mean age: 78.6 years; 98% had a comorbidity.	27	NP and OP samples, collected at two time points, 1 week apart	13	9	RT-PCR Ct values ranged from 13.7 to 37.9 in positive samples
Bullard et al. [25]	All suspected COVID-19 cases had SARS-CoV-2 RT-PCR performed on samples at Cadham Provincial Laboratory. Median age of the patients sampled was 45 years (range: 30–59).	90 (26 with positive viral isolation)	NP and endotracheal samples, from diagnostic samples of individuals who tested positive by RT-PCR from day 0 to 21 post symptom onset	21	8	Positive viral culture samples had lower Ct values than negative cultures (17 (IQR: 16–19) vs 27 (IQR: 22–33)). For every increase in unit in Ct value, the odds of a positive culture decreased by 32%. No growth in samples with Ct > 24.
Decker et al. [21]	62-year-old male heart transplant recipient who was hospitalised with mild disease severity	1	Throat samples, collected serially at 10 time points until day 35 of illness	> 35 (patient still testing positive at study end)	21	Viral culture not successful in samples with RT-PCR Ct > 25
Gautret et al. [29]	Hospitalised patients with age range of 18 to 88 years, 57.5% had at least one comorbidity. Three patients were transferred to ICU, one patient died.	80 (53 with positive viral isolation)	NP samples, collected daily beginning at treatment	12	9	Not reported
Haveri et al. [30]	First COVID-19 case in Finland; hospitalised woman in her 30s from Wuhan with mild disease severity	1	NP samples, collected serially, on days 3, 4, 9, 10, 20 and 23; unclear when viral isolation was attempted	8	4	Ct values on day 4 for different RT-PCR targets: E (29.59), RdRp (30.87), N (31.78)
COVID-19 Investigation Team [26]	Convenience sample of the first 12 US patients confirmed to have COVID-19; five patients had underlying conditions. Median age was 53 years (range: 21–68); mild to moderate illness; seven patients hospitalised but none requiring mechanical ventilation and all showing recovery.	12 (9 with positive viral isolation)	NP and OP samples, taken on days 1–9 from symptom onset; not attempted in later specimens	29	9	Positive viral isolation from samples with RT-PCR Ct values of 12.3–35.7
Lescure et al. [27]	Patients were three men (aged 31 years, 48 years, and 80 years) and two women (aged 30 years and 46 years).	5	NP samples, taken from patients once only at days 2, 2, 6, 7, 9 since symptom onset.	24 (until patient death)	2	Positive viral isolation in samples with RdRp Ct values of 23.6 and 24.4, E gene Ct of 22.8 and 20.0, RdRp IP Ct of 23.0 and 19.3, GAPDH (housekeeping gene) Ct of 26.5 and 25.6
Liu et al. [31]	50-year-old hospitalised woman with mild disease and no comorbidities	1	Throat and sputum samples, collected daily	63	18	Not reported
Million et al. [23]	Hospitalised patients with a mean age of 47.9 (SD 17.5). 973 patients (91.7%) had good clinical outcome; 38 had severe outcomes including death.	1,061 (915 attempted, 204 positive viral isolations, 11 individuals with daily samples)	NP samples, collected daily for 11 participants	> 15 (patient still testing positive at study end)	9	Not reported
Perera et al. [32]	Hospitalised patients positive for COVID-19 with mixed disease severity and an age range of 17–75 years	35	NP, throat, sputum and saliva samples, not collected at predefined intervals; isolation attempted in all positive samples (n = 68)	> 30	8	Not reported
Wölfel et al. [33]	Hospitalised, young-to-middle-aged patients with minimal pre-existing disease and mild symptoms; patients identified based on close contact with an index case and not based on symptoms	9	OP, NP, and sputum samples, collected daily beginning from 2 to 8 days from symptom onset; isolation attempted on multiple occasions from positive samples.	28	8	Not reported
Young et al. [34]	Patients hospitalised with COVID-19 with mixed disease severity, with an age range of 35–56 years. 38% had any comorbidity	100	NP serial samples taken on days 1, 3, 7, 14, 21 and 28 after enrolment; viral culture attempted from samples from 74 patients, but unclear when	48	14	Viral isolation not positive when Ct value was > 30

CPE: cytopathic effect; COVID-19: coronavirus disease; Ct: cycle threshold; E: envelope protein gene; GAPDH: glyceraldehyde-3-phosphate dehydrogenase gene (reference housekeeping gene); IgG: immunoglobulin G; IgM: immunoglobulin M; IQR: interquartile range; N: nucleocapsid protein gene; NP: nasopharyngeal; OP: oropharyngeal; RdRp: RNA-dependent RNA polymerase gene; SARS-CoV-2: severe acute respiratory syndrome coronavirus 2.

Studies are presented in alphabetical order by first author.

### Comparison of maximum duration of positive RT-PCR tests and successful viral isolation reported from studies that measured both

**Figure 2:**
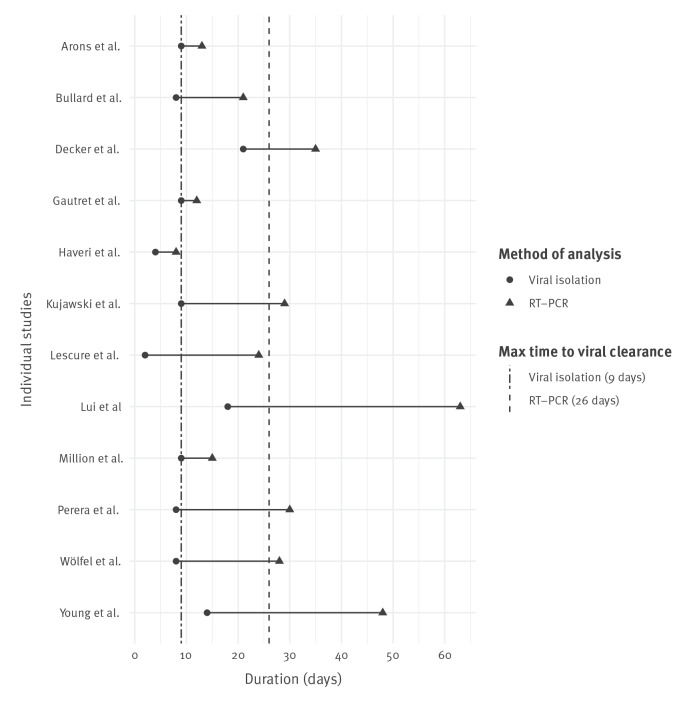
Maximum reported durations of SARS-CoV-2 communicability from studies with data from RT-PCR tests and viral isolation experiments, March–September 2020 (n = 12)

All studies that measured the longest time to viral clearance in both RT-PCR tests and viral isolation experiments reported positive RT-PCR test results after virus was no longer able to be isolated and cultured. The median duration after symptom onset that virus was successfully isolated was 9 days (IQR: 2.25; range: 2–21), while the corresponding median value for longest duration until viral clearance by RT-PCR was 26 days (IQR: 16.8; range: 8–63) (Figure 2). Three studies reported successful viral isolation beyond 10 days (Figure 2).

SARS-CoV-2: severe acute respiratory syndrome coronavirus 2.

Each horizontal line represents data from the same study, with points indicating the longest time to viral clearance reported from each study for viral isolation (circles) and RT-PCR (triangles). Vertical lines represent median reported values across all studies on longest time to viral clearance from viral isolation experiments (dashed and dotted line) and RT-PCR tests (dashed line).

### Maximum reported durations of positive RT-PCR tests and successful viral isolation across all studies

We conducted a final analysis on all included studies that had measurements of maximum duration to viral clearance (n = 142). In 20 studies that successfully achieved viral isolations, viable virus could be isolated from a case across a range of 2–32 days after symptom onset; the median and mean durations of these values across studies were 10.5 days (IQR: 10) and 12.8 days (SD: 7.3), respectively. From the 134 studies with data on maximum duration of positive RT-PCR test results, the shortest reported time until viral clearance was 5 days from symptom onset, while the maximum was 95 days, with respective median and mean values of 25 days (IQR: 19) and 28.8 days (SD: 15.8) (Table 3).

**Table 3:** Data on maximum duration of SARS-CoV-2 communicability from all studies reporting on isolation of live virus and detection of viral nucleic acid by RT-PCR from respiratory samples, March–September 2020 (n = 142)

Method of measurement	Number of included studies	Shortest reported duration	Longest reported duration	Longest reported duration mean (SD)	Longest reported duration median (IQR)
Viral isolation	20	2	32	12.8 (7.3)	10.5 (10)
RT-PCR	134	5	95	28.9 (15.8)	25 (19)

IQR: interquartile range; SARS-CoV-2: severe acute respiratory syndrome coronavirus 2; SD: standard deviation.

Note that some studies provided data for both viral isolation and viral shedding.

## Discussion

This review contributes to the evidence on the period of potential infectivity after infection with SARS-CoV-2, showing that positive detection of viral nucleic acid by RT-PCR can far exceed the duration after which viral transmission may occur. Results from this review suggest that positive RT-PCR results after day 10 following symptom onset of COVID-19 are unlikely to indicate infectiousness. The conclusions of this review are also in line with recently updated guidelines that support relying on symptoms rather than RT-PCR test results for ending isolation precautions in non-healthcare settings [45].

Overall, results from this review concur with previous studies that also support a general 10-day isolation period. However, in reviewing the data from all studies reporting viral isolation, we identified important exceptions. The longest period that virus was isolated after symptom onset was 32 days from a hospitalised patient with severe disease [46]. A duration of 21 days was identified from a report of a single individual with mild symptoms but with significant underlying conditions including a recent heart transplant [21]. A separate case report of an individual without any significant comorbidities and also with mild disease symptoms reported viable virus until 18 days [11]. One case series of 129 hospitalised patients with moderate to severe disease, and mixed degrees of comorbidities, reported a maximum duration of viable virus of 20 days, with a median time to viral clearance in this sample of eight days [38].

Although these exceptions may not occur frequently, they highlight the need to be cautious when COVID-19 cases are being released from isolation into high-risk settings. Previous studies have suggested that there may be differences in duration of viral viability by disease severity with cases of more severe disease potentially having longer infectious viral shedding [7,39]. Despite such trends, the select cases highlighted herein suggest symptom presentation and disease severity do not always follow a pattern with respect to duration of viral viability. Of the studies assessing viral isolation, some also isolated and cultured virus from patients who were asymptomatic or during their convalescent disease period [21-24].

Given the prolonged detection of viral nucleic acid by RT-PCR testing, there have been suggestions to incorporate additional testing metrics when results are being used to inform release from isolation [9,40], such as Ct values. This would be an important and relevant qualification to current protocol especially when considering that positive tests from RT-PCR may persist for several weeks [12-16,41,47]. While the available data on RT-PCR Ct values and viral viability were inconsistent, a number of studies have reported an upper Ct limit for successful viral isolation [9,22,25-28,40,41,46] which may help inform future threshold values.

This review highlights significant heterogeneity in the content and quality of the underlying literature which limits the ability to draw robust inferences to inform isolation protocols from the available data. There was a general paucity of high-quality evidence. We identified few studies that investigated viral isolation and many of the included studies had small sample sizes, making it difficult to draw robust findings from the single subject studies. The included studies suffer from selection bias and lack of generalisability, as case reports and case series often focus on highly specific clinical populations that do not represent the majority of COVID-19 cases. Given that data were often drawn from applied clinical settings, several of the included studies did not follow participants until viral clearance or collect samples at consistent intervals throughout the communicable period. A lack of reporting standards also resulted in incomplete information on valuable metrics such as viral load and Ct values. Finally, although this was not a focus of this review, studies that are able to demonstrate clinical confirmation of transmission are needed to better understand infectivity [47].

This study builds off of previous research by directly comparing results from all studies that measured both the maximum duration of positive RT-PCR test results and longest time to successful viral isolation, thereby adding robustness to interpreting overall findings from the literature. We have made the raw data extracted from all studies in this review available in Supplementary Table S1 for the benefit of other research or public health groups. This research identifies improvements for future research and reporting that should enable more robust syntheses of newly emerging evidence to better inform infection control policies for SARS-CoV-2.

## Supplementary Data

178000 Supplement

## Supplementary Data

61000 SupplementaryTableS1

## Supplementary Data

257000 SupplementaryTableS2
