# Supplementary material for: Determining the communicable period of SARS-CoV-2: A rapid review of the literature, March to September 2020
Source: Euro Surveill. 2021 Apr 8;26(14):2001506. doi: 10.2807/1560-7917.ES.2021.26.14.2001506 (PMC8034061; doi:10.2807/1560-7917.ES.2021.26.14.2001506)
Supplement: Supplement [file 20-01506_PARK_Supplement.pdf]

This supplementary material is hosted by Eurosurveillance as supporting information alongside the article “Determining the communicable period of SARS-CoV-2: A rapid review of the literature, March to September 2020”, on behalf of the authors, who remain responsible for the accuracy and appropriateness of the content. The same standards for ethics, copyright, attributions and permissions as for the article apply. Supplements are not edited by Eurosurveillance and the journal is not responsible for the maintenance of any links or email addresses provided therein.

**Supplement S1.** Detailed information on search strategies and study inclusion criteria.

Appendix A – Search Terms Used:

*1. Viral Clearance/Shedding Searches*

**MEDLINE**

1. ((((exp Coronavirus/ or exp Coronavirus Infections/ or (coronavirus* or corona virus* or OC43 or NL63 or 229E or HKU1 or HCoV* or ncov* or covid* or sars-cov* or sarscov* or Sars-coronavirus* or Severe Acute Respiratory Syndrome Coronavirus*).mp.) and ((20191* or 202*).dp. or 20190101:20301231.(ep).)) not (SARS or SARS-CoV or MERS or MERS-CoV or Middle East respiratory syndrome or camel* or dromedar* or equine or coronary or coronal or covidence* or covidien or influenza virus or HIV or bovine or calves or TGEV or feline or porcine or BCoV or PED or PEDV or PDCoV or FIPV or FCoV or SADS-CoV or canine or CCov or zoonotic or avian influenza or H1N1 or H5N1 or H5N6 or IBV or murine corona*).mp.) or ((((pneumonia or covid* or coronavirus* or corona virus* or ncov* or 2019-ncov or sars*).mp. or exp pneumonia/) and Wuhan.mp.) or (2019-ncov or ncov19 or ncov-19 or 2019-novel CoV or sars-cov2 or sars-cov-2 or sarscov2 or sarscov-2 or Sars-coronavirus2 or Sars-coronavirus-2 or SARS-like coronavirus* or coronavirus-19 or covid19 or covid-19 or covid 2019 or ((novel or new or nouveau) adj2 (CoV on nCoV or covid or coronavirus* or corona virus or Pandemi*2)) or ((covid or covid19 or covid-19) and pandemic*2) or (coronavirus* and pneumonia)).mp. or COVID-19.rx,px,ox. or severe acute respiratory syndrome coronavirus 2.os. or ("32240632" or "32236488" or "32268021" or "32267941" or "32169616" or "32267649" or "32267499" or "32267344" or "32248853" or "32246156" or "32243118" or "32240583" or "32237674" or "32234725" or "32173381" or "32227595" or "32185863" or "32221979" or "32213260" or "32205350" or "32202721" or "32197097" or "32196032" or "32188729" or "32176889" or "32088947" or "32277065" or "32273472" or "32273444" or "32145185" or "31917786" or "32267384" or "32265186" or "32253187" or "32265567" or "32231286" or "32105468" or "32179788" or "32152361" or "32152148" or "32140676" or "32053580" or "32029604" or "32127714" or "32047315" or "32020111" or "32267950" or "32249952" or "32172715").ui.)) and 20191201:20301231.(dt). (8287)
2. (infectious or transmission or transmitted).ti. (130621)
3. ((viral* or virus) adj2 (shed* or clear* or load or dynamics or detect* or assessment or duration)).tw,kf. (54897)
4. ((discharge? or recovered) adj6 positive).tw,kf. (2484)

5. ((communicable or infectious) adj2 period).tw,kf. (496)
6. or/2-5 (185826)
7. 1 and 6 (423)
8. limit 7 to english language (409)

**Embase**

1. ((exp Coronavirinae/ or coronavirus*.mp.) and (wuhan or beijing or shanghai or 2019-nCoV or Covid-19 or SARS-CoV-2).mp.) or ((Coronavirus*.ti. or (novel coronavirus*.mp. and (exp China/ or china.mp.)) or ((pneumonia.mp. or exp pneumonia/) and Wuhan.mp.) or ("Covid-19" or "2019-nCoV" or "SARS-CoV-2").mp. or exp Coronavirus Infection/) and ("2020" or "2021").yr.) (7113)
2. (infectious or transmission or transmitted).ti. (131541)
3. ((viral* or virus) adj2 (shed* or clear* or load or dynamics or detect* or assessment or duration)).tw,kw. (76591)
4. ((discharge? or recovered) adj6 positive).tw,kw. (3547)
5. ((communicable or infectious) adj2 period).tw,kw. (572)
6. or/2-5 (208949)
7. 1 and 6 (411)
8. limit 7 to english language (388)

**Google Scholar**

- Covid AND “viral shedding”
- Covid AND “viral clearance”
- Covid AND transmission
- Covid AND asymptomatic
- Covid AND infectious period

**medRxiv/arXiv**

- Covid viral shedding
- Covid infectious period
- Covid asymptomatic
- Covid “viral clearance”

*2. Viral Isolation/Culture Searches*

**MEDLINE**

Database: Ovid MEDLINE(R) and Epub Ahead of Print, In-Process & Other Non-Indexed Citations, Daily and Versions(R) <1946 to July 01, 2020>

Search Strategy:

--------------------------------------------------------------------------------

1 ((((exp Coronavirus/ or exp Coronavirus Infections/ or (coronavirus* or corona virus* or OC43 or NL63 or 229E or HKU1 or HCoV* or ncov* or covid* or sars-cov* or sarscov* or Sars-coronavirus* or Severe Acute Respiratory Syndrome Coronavirus*).mp.) and ((20191* or 202*).dp. or 20190101:20301231.(ep).)) not (SARS or SARS-CoV or MERS or MERS-CoV or Middle East respiratory syndrome or camel* or dromedar* or equine or coronary or coronal or covidence* or covidien or influenza virus or HIV or bovine or calves or TGEV or feline or porcine or BCoV or PED or PEDV or PDCoV or FIPV or FCoV or SADS-CoV or canine or CCov or zoonotic or avian influenza or H1N1 or H5N1 or H5N6 or IBV or murine corona*).mp.) or ((((pneumonia or covid* or coronavirus* or corona virus* or ncov* or 2019-ncov or sars*).mp. or exp pneumonia/) and Wuhan.mp.) or (2019-ncov or ncov19 or ncov-19 or 2019-novel CoV or sars-cov2 or sars-cov-2 or sarscov2 or sarscov-2 or Sars-coronavirus2 or Sars-coronavirus-2 or SARS-like coronavirus* or coronavirus-19 or covid19 or covid-19 or covid 2019 or ((novel or new or nouveau) adj2 (CoV on nCoV or covid or coronavirus* or corona virus or Pandemi*2)) or ((covid or covid19 or covid-19) and pandemic*2) or (coronavirus* and pneumonia)).mp. or COVID-19.rx,px,ox. or severe acute respiratory syndrome coronavirus 2.os. or ("32240632" or "32236488" or "32268021" or "32267941" or "32169616" or "32267649" or "32267499" or "32267344" or "32248853" or "32246156" or "32243118" or "32240583" or "32237674" or "32234725" or "32173381" or "32227595" or "32185863" or "32221979" or "32213260" or "32205350" or "32202721" or "32197097" or "32196032" or "32188729" or "32176889" or "32088947" or "32277065" or "32273472" or "32273444" or "32145185" or "31917786" or "32267384" or "32265186" or "32253187" or "32265567" or "32231286" or "32105468" or "32179788" or "32152361" or "32152148" or "32140676" or "32053580" or "32029604" or "32127714" or "32047315" or "32020111" or "32267950" or "32249952" or "32172715").ui.)) and 20191201:20301231.(dt). (29800)

2 ((viral* or virus) adj6 (culture? or isolation or isolated)).tw,kf. (42320)

3 1 and 2 (103)

**Embase**

Database: Embase <1974 to 2020 July 01>

Search Strategy:

--------------------------------------------------------------------------------

1 ((exp Coronavirinae/ or coronavirus*.mp.) and (wuhan or beijing or shanghai or 2019-nCoV or Covid-19 or SARS-CoV-2).mp.) or ((Coronavirus*.ti. or (novel coronavirus*.mp. and (exp China/ or china.mp.)) or ((pneumonia.mp. or exp pneumonia/) and Wuhan.mp.) or ("Covid-19" or "2019-nCoV" or "SARS-CoV-2").mp. or exp Coronavirus Infection/) and ("2020" or "2021").yr.) (26353)

2 ((viral* or virus) adj6 (culture? or isolation or isolated)).tw,kw. (41653)

3 1 and 2 (106)

**Google Scholar**

- Covid virus isolation
- Covid viral isolation
- Covid virus culture
- Covid viral culture

**medRxiv/arXiv**

- Title "COVID" (match all words) and abstract or title "viral isolation" (match phrase words)
- Title "COVID" (match all words) and abstract or title "virus isolation" (match phrase words)
- Title "COVID" (match all words) and abstract or title "virus culture" (match phrase words)
- Title "COVID" (match all words) and abstract or title "viral culture" (match phrase words)

Appendix B – Inclusion/Exclusion Criteria:

| Inclusion Criteria | Exclusion Criteria |
|---|---|
| Study had to present empirical data | Commentaries and editorials were excluded.<br><br>Reviews and guidelines were excluded and used for targeted citation chaining. |
| Study was conducted in human populations | In vitro studies excluded |
| Study had to report on duration of transmission or possible infectivity | Studies presenting no information on durations (ie, those on viral kinetics with no durations reported, or on possibility of vertical transmission etc) were excluded.<br><br>Studies reporting on seroconversion were excluded. |
