# Supplementary material for: Determining the communicable period of SARS-CoV-2: A rapid review of the literature, March to September 2020
Source: Euro Surveill. 2021 Apr 8;26(14):2001506. doi: 10.2807/1560-7917.ES.2021.26.14.2001506 (PMC8034061; doi:10.2807/1560-7917.ES.2021.26.14.2001506)
Supplement: SupplementaryTableS2 [file 20-01506_PARK_Supplementary-TableS2.pdf]

This supplementary material is hosted by Eurosurveillance as supporting information alongside the article "Determining the communicable period of SARS-CoV-2: A rapid review of the literature, March to September 2020", on behalf of the authors, who remain responsible for the accuracy and appropriateness of the content. The same standards for ethics, copyright, attributions and permissions as for the article apply. Supplements are not edited by Eurosurveillance and the journal is not responsible for the maintenance of any links or email addresses provided therein.

Supplementary Table S2. **Detailed data from papers investigating durations until viral isolation and culture.** Papers are presented in alphabetical order by first author.

**First Author**<br><br>**Journal & publication date/status**	**Country**	**Study design**	**Study population**	**Sample Size**	**Sample types taken for isolation, sampling method**	**Viral shedding (max)**	**Viral isolation (max)**	**Notable findings**	**Symptoms**
Arons et al. (1)<br><br>*New England Journal of Medicine (April 24, 2020)*	United States	Serial cross-sectional	Patients in a skilled nursing facility with mixed disease severity, a mean age of 78.6, 98% had a co-morbidity	48 (27 individuals with 2 samples taken)	NP & OP samples, collected at 2 time points, 1 week apart.	13 days	9 days	Positive cultures in 17 out of 24 pre-symptomatic patents and 1 out of 3 asymptomatic patients.<br><br>Viable virus isolated up to 6 days before onset of symptoms. RT-PCR positive up to 7 days prior to symptom onset.<br><br>RT-PCR Ct values ranged from 13.7-37.9 in positive samples.	Includes asymptomatic and pre-symptomatic patients. Most common new symptoms were fever, cough and malaise.

Basile et al. (2)	Australia	Case series	Patients with positive PCR test, mixed disease severity (ICU, admitted and outpatients), a mean age of 40 years (8-78 years), 75% M	195 patients (234 samples)	NP, Nose & throat, sputum, ETA, nbBAL, at mean 11 days (up to 29 days) after symptom onset	N/A	18 days	Ct values mean=34, IQR: 29-39 (range: 17.5 to 40)<br><br>Mean Ct across all cultures increased with time from symptom onset.<br><br>Positive viral culture samples collected closer to symptom onset date than were negative samples.	Types of symptoms not described.
Bullard et al. (3)<br><br>*Clinical Infectious Diseases (May 22, 2020)*	Canada	Cross-sectional	All samples in this study were obtained to support routine care and surveillance of the public health response in the province of Manitoba, Canada. All suspect COVID-19 cases had SARS-CoV-2 RT-PCR performed on nasopharyngeal (NP) or endotracheal (ETT) samples at Cadham Provincial Laboratory. Median age of the patients sampled was 45 (range: 30-59 years). Forty nine percent of samples were from males.	90 (26 with positive viral isolation)	NP and endotracheal samples, from diagnostic samples of individuals who tested positive by RT-PCR from day 0 to 21 post symptom onset	21 days	8 days	Positive viral culture samples had significantly lower Ct values than negative cultures (17 (16-19) vs 27 (22-33)). For every increase in unit in Ct value, the odds of a positive culture decreased by 32%.<br><br>Time from symptom to test time was significantly lower in positive vs negative cultures (3 (2-4) vs 7 (2-11)). For every day since symptom the odds of a positive culture decreased by 37%.<br><br>Probability of a successful positive culture peaked on day 3 and decreased after that point.<br><br>No growth in samples with Ct >24.	Not described.

Chang et al. (4)<br><br>*The Journal of Allergy and Clinical Immunology in Practice (June 20, 2020)*	China	Case series	Hospitalized patients with mixed disease severity, who re-tested positive after discharge. 3 required ICU admission.	69 (4 attempted and 0 positive viral isolations)	Throat samples, collected serially while in hospital and after discharge. Only attempted viral isolation from samples taken during the convalescent period, sent while patients were at home.	57 days	N/A	No positive viral isolation/ culture during convalescent period.	Fever present in 82% of patients, cough in 60% of patients, sputum in 25%. 2 patients were asymptomatic.
Decker et al. (5)<br><br>*American Journal of Transplantation (June, 2020)*	Germany	Case report	62 year old male heart transplant recipient who was hospitalized with mild disease severity	1	Throat samples, collected serially at 10 time points until day 35 of illness.	>35 days (patient still testing positive at study end)	21 days	Patient post-symptomatic at time of positive viral cultures.<br><br>Viral culture not successful in samples with PT-PCR Ct >25 (log 5.3 copies/ml).	Fever on day 1 that resolved within 12 hours, second fever that spiked and resolved at day 7. Other symptoms were mild rhinorrhea and impaired exercise capacity.

Folgueira et al. (6)<br><br>*MedrXiv** Pre-print *(June 12, 2020)*	Spain	Cross-sectional	Patients with mixed disease severity. Patients with mild symptoms (n=24) were healthcare workers, largely women, and assessed in outpatient settings. Patients with severe disease were hospitalized (n=41), of which 5 died.	65 (106 samples taken)	NP samples and bronchial aspirates, from diagnostic samples of individuals diagnosed as outpatients and those followed up for hospital care. Median time of sample collection for hospitalized patients was 19.6 days and for outpatients was 16.5 days.	32 days	N/A	Positive viral culture was obtained from a higher proportion of patients with severe disease vs mild symptoms (53.4% of samples vs 36.0%). All samples from deceased patients were able to be cultured.<br><br>Samples with Ct values < 25 had >90% positive viral culture. However, samples with low viral loads (Ct > 35) could still harbor viable virus.	6 hospitalized patients were admitted to the ICU and required mechanical ventilation. 5 patients died.
Gautret et al. (7)<br><br>*Travel Medicine and Infectious diseases (April 2020)*	France	Case series	Hospitalized patients with age range of 18 to 88 years, 57.5% had at least one comorbidity. 3 patients were transferred to ICU, 1 patients died.	80 (53 with positive viral isolation)	NP samples, collected daily beginning at treatment.	12 days	9 days	Viral cultures were negative in 97.5% of patients at day 5.	4 patients were asymptomatic, 14 patients had fever as a symptom, 33 had upper respiratory tract symptoms and 43 had lower respiratory tract symptoms.

Gniazdowski et al. (8)<br><br>*MedrXiv**Pre-print (August 6, 2020)	United States	Case series	Patients with COVID-19 with mixed disease severity, with an age range of 4-93 years.	29	NP samples collected as a part of standard of care testing.	NA	22 days	Mean Ct value for infectious virus was 18.8 ± 3.4, which was significantly lower than the mean of non-infectious virus of 27.1 ± 5.7 ($p<0.0001$). 28.6% of cultures were negative yet they had the same Ct range as positive cultures and 11.9% had a Ct value of <20.<br><br>Duration of infectious virus not associated with severity of illness, but with the duration of symptoms in most patients.	Symptoms reported by patients were fever (67%), cough (54%), dyspnea (39%), myalgia (30%) and gastrointestinal (28%).
Haveri et al. (9)<br><br>*Euro Surveillance (Mar 25, 2020)*	Finland	Case report	First COVID-19 case in Finland. Hospitalized woman in her 30s from Wuhan with mild disease severity.	1	NP samples, collected serially, on days 3, 4, 9, 10, 20 and 23, unclear when viral isolation was attempted.	8 days	4 days	Showed late seroconversion: Antibodies were undetectable on Day 4 after onset of symptoms,IgG titres rose to 80 and 1,280 and IgM titres to 80 and 320 on Days 9 and 20.<br><br>Ct values on day 4 for different RT-PCR targets: E (29.59), RdRp (30.87), N (31.78).	Respiratory symptoms on onset, followed by high fever on day 2, post-symptomatic by day 7.

Jeong et al. (10)<br><br>*Clinical Microbiology & Infection (July 23, 2020)*	South Korea	Case series	Hospitalized patients with mixed disease severity, with range of ages of 51-63 years.	5	NP, OP or saliva samples collected on days 8, 11, 13, 15 and 30.	NA	15 days	Positive cultures from saliva samples.	Not described.
Kim et al. (11)<br><br>*J Korean Med Sci (Feb 24, 2020)*	South Korea	Case series	First 2 patients with COVID-19 in South Korea. 1 35-year old woman and 1 55-year old man, both with mild symptoms.	2	Respiratory samples, collected daily starting day 2 and day 14 of illness.	26 days	N/A	No positive viral isolation/ culture.<br><br>Upper respiratory RdRp Ct values ranged from 25.05-36.69 and lower respiratory RdRp Ct values ranged from 22.05-32.63.	Patient 1 had fever at onset of illness until day 9, nasal congestion on days 6-8, dyspnea on days 9-11, cough on days 7-13, sputum on days 9-13 and mild loose stools on days 4-19. Patient 2 had sore throat from illness onset until day 17, fever beginning around day 10 and lasting until 16, cough on days 16-18 and loose stool on days 19-21.
Kim et al. (12)<br><br>Osong Public Health & Research Perspectives	South Korea	Case series	Hospitalized patients aged 9-80 years	22	NP, OP and sputum samples taken serially	17	N/A	No positive viral isolation/ culture	Not described.

Kujawski et al. (13)<br><br>*Nature (June 26, 2020)*	United States	Case series	Convenience sample of the first 12 US patients confirmed to have COVID-19. 5 patients had underlying conditions. Median age was 53 years (range: 21–68); 8 patients were male. Had mild to moderate illness with 7 patients hospitalized but none requiring mechanical ventilation and all showing recovery.	12 (9 with positive viral isolation)	NP and OP samples, which were taken on days 1-9 from symptom onset. Not attempted in later specimens.	29 days	9 days	Positive viral isolation from samples with RT-PCR Ct values of 12.3–35.7.	Median symptom duration of 14 days, cough reported as last symptom to resolve. Median duration of fever was 9 days (range 2-11), with a peak body temperature at median 9 days (range 4-10)
Kumar et al. (14)<br><br>*Canadian Medical Association Journal (April 24, 2020)*	Canada	Case report	76 year old man with multiple comorbidities, who initially tested negative for COVID-19.	1	NP sample, collected on day 4 of hospital admission (which was 11 days after exposure, and day 6 after cough worsened)	17 days	N/A	No positive viral isolation/ culture.<br><br>Sputum culture conducted on day 4, which is the same day that patient tested negative for SARS-CoV-2 in a NP swab. NP swab, positive on day 7 of admission (18 days after exposure; 13 days after cough worsened)	Worsening cough on days 1-6, fatigue, exertional dyspnea, fevers, low appetite and diarrhea on days 2-6
Ladhani et al. (15)<br><br>*EClinicalMedicine (Sept 9, 2020)*	United Kingdom	Case series	Residents and staff at care homes in UK with suspected COVID-19 outbreaks, with a median age of 85 (IQR 78-90) for residents and for staff median age was 47 (IQR 35-56).	518	NP samples taken as a part of testing during outbreak.	NA	13 days	No difference in Ct found for positive tests where the patient was pre-symptomatic, symptomatic or post-symptomatic.<br><br>Differences in ability to isolate virus found for Ct values < 20 (100%) vs Ct 30 – 35 (17%)	Not described.

Lescure et al. (16)<br><br>*Lancet Infectious Diseases (Mar 27, 2020)*	France	Case series	Patients were three men (aged 31 years, 48 years, and 80 years) and two women (aged 30 years and 46 years).	5	NP samples, taken from patients once only at days 2, 2, 6, 7, 9 since symptom onset.	24 days (until patient death)	2 days	Positive viral isolation in samples with RdRp Ct values of 23.6 and 24.4, E gene Ct of 22.8 and 20.0, RdRp IP Ct of 23.0 and 19.3, GAPDH (housekeeping gene) Ct of 26.5 and 25.6.<br><br>Positive isolate titre was 6.25×10^5 and 3.0×10^7.	Describes 3 subtypes of symptom progression: first, mild cases through two paucisymptomatic patients aged younger than 50 years who were diagnosed early, with high viral load in nasopharyngeal samples, suggesting a significant shedding of SARS-CoV-2, reflected by virus detection by RT-PCR; second, two young patients presenting with mild symptoms at admission and experiencing a secondary progression to pneumonia and severe disease by days 10–11; and third, an older patient with a rapid evolution towards critical disease with multiple organ failure and a long and sustained persistence of SARS-CoV-2 nasopharyngeal detection associated with viral RNA detection in multiple sites

L'Huillier et al (17)<br><br>*Emerging Infectious Diseases (June 30, 2020)*	Switzerland	Case series	Children with mild disease. Isolated virus from children of all ages; the youngest was 7 days of age	23 (12 with positive viral isolation)	NP samples, taken from patients at days 0 - 5 after symptom onset. Did not attempt after day 5.	N/A	5 days	Median viral load was higher for patients with positive viral isolation than for those without isolation. ($1.7x10^{8}$ vs $6.9x10^{3}$).	Symptoms did not differ between children from whom a positive viral isolation was obtained vs not.
Liu et al. (18)<br><br>*The Journal of Infection (April 18, 2020)*	Taiwan	Case report	50 year old hospitalized woman with mild disease and no comorbidities	1	Throat and sputum samples, collected daily.	63 days	18 days	Viral cultures positive after resolution of symptoms. Antibodies detected on day 10, but viral cultures remained positive until day 18.<br><br>Virus isolated from throat swabs at admission, and from sputum until 18 days after symptom onset.	Fever resolved on day 10.
Million et al. (19)<br><br>*Travel Medicine & Infectious Disease (May 2020)*	France	Case series	Hospitalized patients with a mean age of 47.9 (SD 17.5), 2.6% had cancer, 7.4% had diabetes, 4.3% had coronary artery disease, 14% had hypertension, 10.5% had chronic respiratory disease and 5.8% had obesity. 973 patients (91.7% had good clinical outcome) with 38 severe outcomes including death.	1061 (915 attempted, 204 positive viral isolations, 11 individuals with daily samples)	NP samples, collected daily for 11 participants.	>15 days (patient still testing positive at study end)	9 days	Prolonged viral shedding associated with high viral load at diagnosis, but no positive viral cultures after day 9.	Not described.

Perera et al. (20)<br><br>*Emerging Infectious Diseases (Nov 2020)*	Hong Kong	Case series	Hospitalized patients positive for COVID-19 with mixed disease severity and an age range of 17-75 years.	35	NP, throat, sputum and saliva samples, not collected at predefined intervals.	>30 days	8 days	Viral load associated with positive cultures (12/17 for specimens with viral loads ≥7.0 log10 copies/mL, 3/11 for specimens with viral loads 6.0–6.99 log10 copies/mL, 1/7 specimens with viral loads 5.0–5.99 log10 copies/mL, and 0/33 specimens viral loads <5 log10 copies/mL)	Not described.
Singanayagam et al. (21)<br><br>*Eurosurveillance*	United Kingdom	Case series	Symptomatic cases that tested positive and had a clear record of dates of symptom onset and sample collection.	176	Upper respiratory samples from routine testing, not collected at pre-defined intervals.	NA	12 days	Odds ratio of positive viral culture decreased by 0.67 with each increase of Ct value (95% CI: 0.58–0.77).<br><br>In 5 symptomatic cases with non-severe illness were able to isolate virus from samples with Ct >35.<br><br>Positive culture rate was significantly higher during week 1 than week 2 (74% vs 20%; p = 0.002), with the probability of a positive culture at 10 days after symptom onset being 6% (95% CI: 0.9–31.2%).<br><br>No difference in Ct values or positive cultures between different age groups.	Not described.

Sun et al. (22)<br><br>*Emerging Microbes & Infections*	China	Case report	72-year old man admitted to hospital	1	OP samples taken serially at unclear intervals	42	N/A	No positive viral isolation/ culture	Patient had cough and fever when admitted to hospital. His condition deteriorated and he was ventilated.
van Kampen et al. (23)<br><br>*MedrXiv**Pre-print *(June 9, 2020)*	The Netherlands	Case series	Hospitalized patients with severe or critical COVID-19, admitted to medium acute care (40, 31%) or to intensive care (89, 69%). 11 patients were severely immunocompromised and 19 were non-severely immunocompromised.	129 (23 individuals with positive viral isolations)	Upper respiratory and sputum samples, collected serially from diagnostic samples only, not at pre-defined intervals.	N/A	20 days	Median duration of infectious virus shedding was 8 days (IQR 5 – 11). ≤5% probability of isolating infectious virus when duration of symptoms was 15.3 days or more (95% CI 13.2-17.2)<br><br><5% probability of isolation when viral load went below 6.51 log10 RNA copies per mL.<br><br>Viral load was associated with infectious viral shedding; median viral load was significantly higher in culture positive samples than in culture negative samples.<br><br>Shedding of infectious virus dropped rapidly to undetectable levels upon seroconversion.	Assumption that all patients with positive viral isolation were symptomatic at the time of sampling, given the patient population.

Wölfel et al. (24)<br><br>*Nature (April 1, 2020)*	Germany	Case series	Hospitalized, young-to-middle aged patients with minimal pre-existing disease (one each of hypothyroidism, COPD, hypercholesteremia), and relatively mild symptoms. Patients identified based on close contact with an index case and not based on symptoms.	9	OP, NP, and sputum samples, collected daily beginning from 2-8 days from symptom onset. Isolation attempted on multiple occasions from positive samples.	28 daysdid not	8 days	Peak viral shedding in upper respiratory tract during first 5 days, with prolonged shedding in sputum. Sputum samples remained RNA-positive over three weeks in six of the nine patients, despite full resolution of symptoms.<br><br>Successful viral isolation found before and after seroconversion.<br><br>Threshold of at least 100,000 viral RNA copies per ml of sputum required for isolation.<br><br>Positive rate of virus isolation in first week of symptoms was 16.66% in NP/OP swab samples and 83.33% in sputum samples, but after day 8 no positive isolates were obtained despite ongoing high viral loads.<br><br>Seroconversion associated with a steady, not abrupt, decline of viral load. Seroconversion occurred for all patients by day 14.	1 patient had fever as an initial symptom and 3 patients showed fever as a later symptom and the fever resolved in these 3 patients on day 4, day 4 and day 8. 1 patient was asymptomatic.

Young et al. (25)<br><br>*Clinical Infectious Diseases (Aug 28, 2020)*	Singapor e	Case series	Patients hospitalized with COVID-19 with mixed disease severity, with an age range of 35-56. 38% had any comorbidity; 10% with diabetes and 19% with hypertension.	100	NP serial samples taken on days 1, 3, 7, 14, 21 and 28 after enrollment.	48 days	14 days	Viral isolation not positive when Ct value was >30.<br><br>No association found for duration of viral shedding when stratified for disease severity.<br><br>No correlation between viral isolation and infection severity, demographics or symptoms.	Presenting symptoms: 76% had fever, 70% had cough, 47% had sore throat and/or rhinorrhea, 19% had diarrhea, and 17% had dyspnea.
Ct, cycle threshold; E, envelope protein gene; GAPDH, glyceraldehyde-3-phosphate dehydrogenase gene (reference housekeeping gene); IgG, immunoglobulin G; IgM, immunoglobulin M; N, nucleocapsid protein gene; NP, nasopharyngeal; OP, oropharyngeal; RdRp, RNA-dependent RNA polymerase gene									

References

1. Arons MM, Hatfield KM, Reddy SC, Kimball A, James A, Jacobs JR, et al. Presymptomatic SARS-CoV-2 Infections and Transmission in a Skilled Nursing Facility. N Engl J Med. 2020 Apr 24;24:24.

2. Basile K, McPhie K, Carter I, Alderson S, Rahman H, Donovan L, et al. Cell-based culture of SARS-CoV-2 informs infectivity and safe de-isolation assessments during COVID-19. Clinical Infectious Diseases [Internet]. 2020 Oct 24 [cited 2020 Dec 7];(ciaa1579). Available from: https://doi.org/10.1093/cid/ciaa1579

3. Bullard J, Dust K, Funk D, Strong JE, Alexander D, Garnett L, et al. Predicting infectious SARS-CoV-2 from diagnostic samples. Clin Infect Dis [Internet]. [cited 2020 Jul 20]; Available from: https://academic.oup.com/cid/article/doi/10.1093/cid/ciaa638/5842165

4. Chang, Zhao P, Zhang DW, Dong JH, Xu Z, Yang G, et al. Persistent Viral Presence Determines the Clinical Course of the Disease in COVID-19. J Allergy Clin Immunol Pract. 2020 Jun 20;20:20.

5. Decker A, Welzel M, Laubner K, Grundmann S, Kochs G, Panning M, et al. Prolonged SARS-CoV-2 shedding and mild course of COVID-19 in a patient after recent heart transplantation. Am J Transplant. 2020 Jun;09:09.

6. Folgueira MD, Luczkowiak J, Lasala F, Perez-Rivilla A, Delgado R. Persistent SARS-CoV-2 replication in severe COVID-19. medRxiv. 2020 Jun 12;2020.06.10.20127837.

7. Gautret P, Lagier J-C, Parola P, Meddeb L, Sevestre J, Mailhe M, et al. Clinical and microbiological effect of a combination of hydroxychloroquine and azithromycin in 80 COVID-19 patients with at least a six-day follow up: A pilot observational study. Travel medicine and infectious disease. 2020;101663.

8. Gniazdowski V, Morris CP, Wohl S, Mehoke T, Ramakrishnan S, Thielen P, et al. Repeat COVID-19 Molecular Testing: Correlation with Recovery of Infectious Virus, Molecular Assay Cycle Thresholds, and Analytical Sensitivity. medRxiv. 2020 Aug 6;2020.08.05.20168963.

9. Haveri A, Smura T, Kuivanen S, Osterlund P, Hepojoki J, Ikonen N, et al. Serological and molecular findings during SARS-CoV-2 infection: the first case study in Finland, January to February 2020. Euro Surveill. 3;25(11):03.

10. Jeong HW, Kim S-M, Kim H-S, Kim Y-I, Kim JH, Cho JY, et al. Viable SARS-CoV-2 in various specimens from COVID-19 patients. Clin Microbiol Infect. 2020 Nov;26(11):1520–4.

11. Kim JY, Ko JH, Kim Y, Kim YJ, Kim JM, Chung YS, et al. Viral Load Kinetics of SARS-CoV-2 Infection in First Two Patients in Korea. J Korean Med Sci. 2020 Feb 24;35(7):e86.

12. Kim JM, Kim HM, Lee EJ, Jo HJ, Yoon Y, Lee NJ, et al. Detection and Isolation of SARS-CoV-2 in Serum, Urine, and Stool Specimens of COVID-19 Patients from the Republic of Korea. Osong Public Health Res Perspect. 2020 Jun;11(3):112-117. https://doi.org/10.24171/j.phrp.2020.11.3.02.

13. Kujawski SA, Wong KK, Collins JP, Epstein L, Killerby ME, Midgley CM, et al. Clinical and virologic characteristics of the first 12 patients with coronavirus disease 2019 (COVID-19) in the United States. Nature Medicine. 2020 Jun;26(6):861–8.

14. Kumar DS, O'Neill SB, Johnston JC, Grant JM, Sweet DD. SARS-CoV-2 infection in a 76-year-old man with negative results for nasopharyngeal swabs and possible nosocomial transmission. Cmaj. 2020 Apr 24;24:24.

15. Ladhani SN, Chow JY, Janarthanan R, Fok J, Crawley-Boevey E, Vusirikala A, et al. Investigation of SARS-CoV-2 outbreaks in six care homes in London, April 2020. EClinicalMedicine [Internet]. 2020 Sep 1 [cited 2020 Dec 7];26. Available from: https://www.thelancet.com/journals/eclinm/article/PIIS2589-5370(20)30277-7/abstract

16. Lescure FX, Bouadma L, Nguyen D, Parisey M, Wicky PH, Behillil S, et al. Clinical and virological data of the first cases of COVID-19 in Europe: a case series. Lancet Infect Dis. 2020 Mar 27;27:27.

17. L'Huillier AG, Torriani G, Pigny F, Kaiser L, Eckerle I. Culture-Competent SARS-CoV-2 in Nasopharynx of Symptomatic Neonates, Children, and Adolescents. Emerg Infect Dis. 2020 Jun 30;26(10):30.

18. Liu W-D, Chang S-Y, Wang J-T, Tsai M-J, Hung C-C, Hsu C-L, et al. Prolonged Virus Shedding Even after Seroconversion in a Patient with COVID-19. J Infect. 2020;(ig9, 7908424).

19. Million M, Lagier JC, Gautret P, Colson P, Fournier PE, Amrane S, et al. Early treatment of COVID-19 patients with hydroxychloroquine and azithromycin: A retrospective analysis of 1061 cases in Marseille, France. Travel Med Infect Dis. 2020 May;101738.

20. Perera RAPM, Tso E, Tsang OTY, Tsang DNC, Fung K, Leung YWY, et al. SARS-CoV-2 Virus Culture and Subgenomic RNA for Respiratory Specimens from Patients with Mild Coronavirus Disease - Volume 26, Number 11—November 2020 - Emerging Infectious Diseases journal - CDC. [cited 2020 Dec 7]; Available from: https://wwwnc.cdc.gov/eid/article/26/11/20-3219_article

21. Singanayagam A, Patel M, Charlett A, Bernal JL, Saliba V, Ellis J, et al. Duration of infectiousness and correlation with RT-PCR cycle threshold values in cases of COVID-19, England, January to May 2020. Eurosurveillance. 2020 Aug 13;25(32):2001483.

22. Sun J, Zhu A, Li H, Zheng K, Zhuang Z, Chen Z, et al. Isolation of infectious SARS-CoV-2 from urine of a COVID-19 patient. Emerg Microbes Infect. 2020 Dec;9(1):991-993. https://doi.org/10.1080/22221751.2020.1760144.

23. van Kampen JJA van, Vijver DAMC van de, Fraaij PLA, Haagmans BL, Lamers MM, Okba N, et al. Shedding of infectious virus in hospitalized patients with coronavirus disease-2019 (COVID-19): duration and key determinants. medRxiv. 2020 Jun 9;2020.06.08.20125310.

24. Wölfel R, Corman VM, Guggemos W, Seilmaier M, Zange S, Müller MA, et al. Virological assessment of hospitalized patients with COVID-2019. Nature. 2020;581(7809):465–9.

25. Young BE, Ong SWX, Ng LFP, Anderson DE, Chia WN, Chia PY, et al. Viral Dynamics and Immune Correlates of Coronavirus Disease 2019 (COVID-19) Severity. Clinical Infectious Diseases [Internet]. 2020 Aug 28 [cited 2020 Dec 7];(ciaa1280). Available from: https://doi.org/10.1093/cid/ciaa1280
